# Interactions Between Cationic Micellar Solution and Aromatic Hydrotropes with Subtle Structural Variations

**DOI:** 10.3390/molecules29225482

**Published:** 2024-11-20

**Authors:** Bin Liu, Shuo Yin, Xia Wu, Xilian Wei, Huifang Xu, Jing Li, Dongmei Lv

**Affiliations:** 1 School of Chemistry and Chemical Engineering, Liaocheng University, Liaocheng 252059, China; binliu@lcu.edu.cn (B.L.); weixilian@126.com (X.W.); 2 College of Pharmacy, Henan University of Chinese Medicine, Zhengzhou 450046, China

**Keywords:** wormlike micelles, hydrotropes, viscoelasticity, rheology, phase behavior

## Abstract

Wormlike micelles (WLMs) with tunable viscoelastic characteristics have emerged as indispensable smart materials with a wide range of applications, which have garnered intense interest over the past few decades. However, quantitatively predicting the effect of various hydrotropes on the rheological behaviors of WLMs remains a challenge. In this article, micelles were formed in a mixture of 3-hexadecyloxy-2-hydroxypropyltrimethylammonium bromide (R_16_HTAB) and aromatic hydrotropes (e.g., sodium benzoate, sodium cinnamate and their derivatives, respectively) in an aqueous solution. The phase behavior, viscoelasticity and thickening mechanism were systematically studied by macroscopic observation, rheological measurements, electrostatic potential analysis and cryogenic transmission electron microscopy (Cryo-TEM). Rheological measurements were used to probe the remarkable viscoelastic properties of micelles stemming from their lengthening and entanglement under the interaction between R_16_HTAB and hydrotropes with structural variations. For an equimolar system of R_16_HTAB and cosolute (40 mM), the relaxation time decreases in the following order: SpMB > SoHB > S4MS > SmMB > S5MS > SB > SmHB > SoMB > SpHB. These results allow us to predict the possible rules for the self-assembly of R_16_HTAB and aromatic hydrotropes, which is conductive to directionally designing and synthesizing smart wormlike micelles.

## 1. Introduction

Above the critical micelle concentration (CMC), surfactant molecules can self-assemble in aqueous solutions into a variety of microstructures like micelles (spherical, rodlike or wormlike), vesicles, lamellae, and columnar and cubic mesophases [1,2]. The formation of these microstructures can be explained by the concept of a molecular packing parameter [1,2], which takes into account the geometric characteristics of the surfactant to estimate the optimal shape of the congeries into which they can pack. Among these microstructures, wormlike micelles have always been a research focus due to their excellent viscoelastic properties and potential applications such as thickeners in liquid dishwashing detergents, consumer products and personal care products [3,4], drag reduction agents in large heating and cooling installations [5,6], fracturing fluids used in oil fields [7,8], soft templates for synthesis of nanomaterials [9], and sieving matrices for DNA separation [10]. In general, alkyl quaternary ammonium salt cationic surfactants (classic specimen is CTAB) are commonly used to construct wormlike micelles with some additive salts, such as inorganic (haloids) [11,12] and organic salts (salicylates or their derivatives) [13,14,15,16]. Compared with the high-concentration necessity of single surfactants to form wormlike micelles, the mixtures of surfactants and additives have much better surface and bulk properties. From an industrial point of view, it is much more economical [17,18] because of the high cost of mass production and purification of surfactant. As a result, surfactant–counterion mixtures are broadly used in many industrial sectors. In addition, if the additive contains stimulus-responsive groups, the mixed solutions can also form an intelligent wormlike micellar system [19] that can trigger the transformation of aggregate structures under various environmental factors, such as pH [20], light [21], electricity [22] and temperature [23], which endow wormlike micelles great application prospects. It is well known that the aggregation patterns of surfactant micellar aqueous solution are very sensitive to the presence of counterion. Small changes in the concentration and structure of counterions can greatly change the length and flexibility of micelles, thus changing the macro-rheological properties. These behaviors are related to the dissolution degree and bonding mode of counterions in micelles. On one hand, the electrostatic interactions between the head groups of surfactants and the counterions make them closer, and counterions might be inserted into the palisade layer of the micelles under the driving of the hydrophobic force; on the other hand, hydrogen bonds could play a crucial role between the molecules. These all lead to the one-dimensional growth of the micelles, thereby enhancing the viscoelasticity of the system. Thus, rheological techniques can be used to investigate the viscoelastic behavior and microstructure change of the wormlike micellar solution.

We know that aromatic salts are the most commonly used anion additives for the formation of wormlike micelles with cationic surfactants. Although much work has been performed on mixtures of quaternary ammonium cationic surfactants with a range of aromatic salts, the exact role of the molecular structure of different aromatic co-solutes in determining interfacial packing is not well understood, and the effect of different substituents on the rheological behaviors of wormlike micelles still need further study. In this paper, mixed micellar solutions were constructed with a cationic surfactant 3-hexadecyloxy-2-hydroxypropyl trimethyl ammonium bromide (R_16_HTAB) as a host compound and a range of derivatives of benzoate and cinnamate as additives. The aim of the study was to investigate and understand the influence of structural change of different additives, such as the substitution of hydrogen on the aromatic ring of sodium benzoate by hydroxyl, methyl and methoxy group, the distance between the substituent groups and the carboxyl group, and the electrical properties in the aromatic cosolute on the self-assembly and viscoelastic behavior of wormlike micelles using macroscopic observation, rheological measurements, electrostatic potential analysis and the Cryo-TEM technique. And the results were rationalized by plotting the electronic density of the additives using computational chemistry and analyzing the relationship between polarity of substituent groups on the aromatic ring and macroscopic behavior. R_16_HTAB is a derivative of CTAB, with a 2-hydroxypropoxy group innovatively inserted at the junction between the head-group and the alkyl chain of CTAB, showing good surface activity and bacteriostatic activity, and non-toxic property [24,25,26,27,28], which greatly broadens its application in different industries field. We expect that these research results will play important roles in the construction of novel self-assembled structures of mixed solutions of cationic surfactants with additives and satisfy actual use in oil fields, medicine and household chemicals for the future.

## 2. Results and Discussion

### 2.1. Benzoate Derivatives Induced Transformation of Micellar Structure of the R_16_HTAB Aqueous Solution

**Scheme 1:**
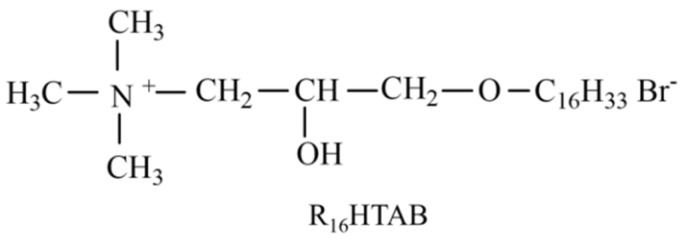
Molecular structure of R_16_HTAB.

**Scheme 2:**
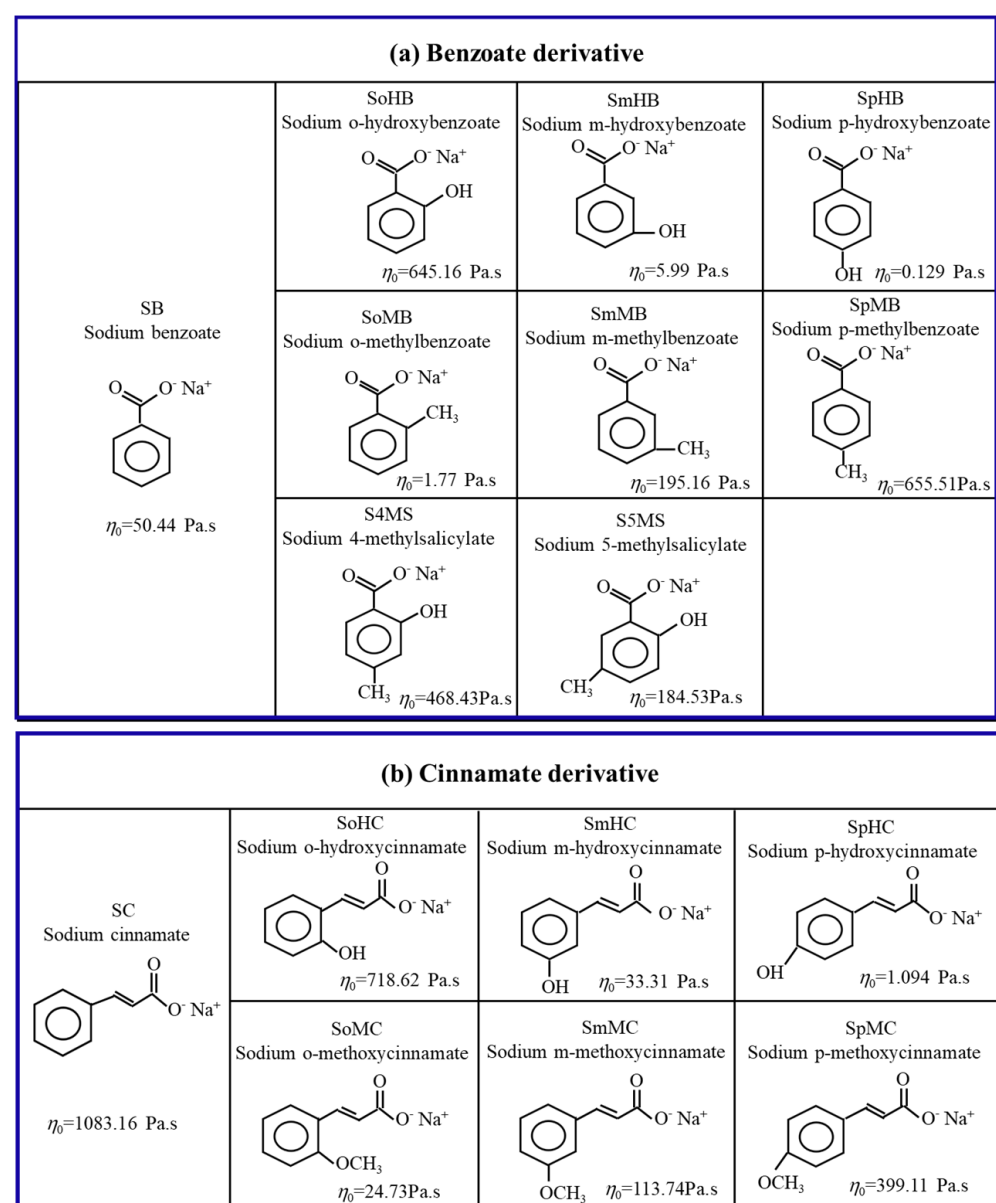
Molecular structures of various hydrotropes with values of the zero-shear Viscositys ($\eta_{0}$) for 40 mM equimolar mixtures of R_16_HTAB and hydrotropes.

**Figure 1:**
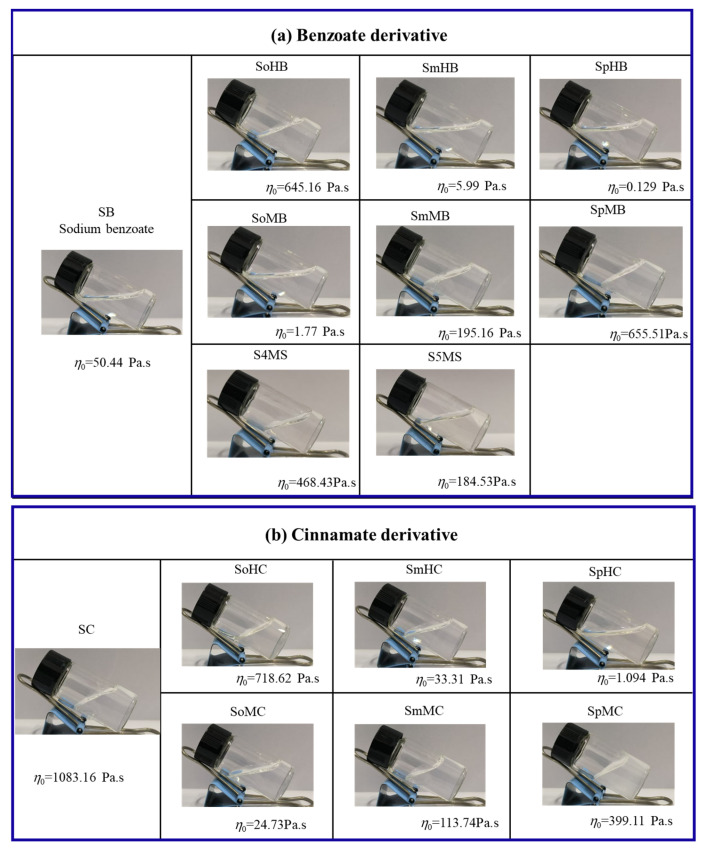
Macroscopic appearance of mixture of R_16_HTAB and different hydrotropes shown in Scheme 2.

Viscosity measurements were performed on equimolar solutions of R_16_HTAB (40 mM) shown in in Scheme 1 and benzoate derivatives in Scheme 2, respectively, because a strong influence, like high viscosity systems, is typically found for mixed solutions when approaching their charge equimolarity. Figure 1 shows apparent states of a series of samples of R_16_HTAB and aromatic hydrotropes. It can be seen that different additives show a great difference in their ability to increase viscosity of cationic surfactants. When the vials were tilted, their flow behavior showed different changes under gravitational force; their behavior changed from more solid-like to more liquid-like (from left to right) in the following sequences: SoHB > SmHB > SpHB; SpMB > SmMB > SoMB; S4MS > S5MS; SoHC > SmHC > SpHC; SpMC > SmMC > SoMC. These results clearly confirm that small variations in the properties and positions of substituents on the benzene ring result in very different macroscopic responses in mixtures with R_16_HTAB.

**Figure 2:**
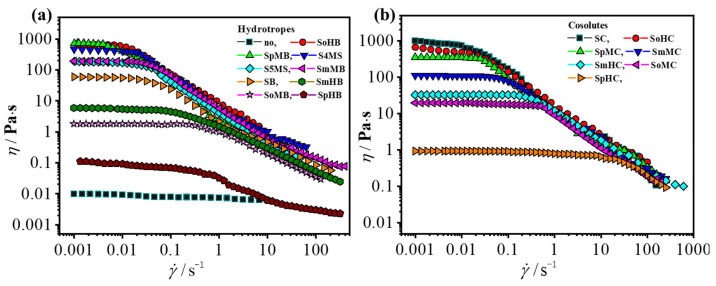
(**a**) Curves of apparent viscosity (*η*) versus shear rate ($\dot{\gamma }$) for the aqueous solutions of R_16_HTAB/ benzoate derivatives and (**b**) R_16_HTAB/ cinnamate derivatives.

Figure 2 shows the steady-shear viscosity curves of different samples shown in Figure 1 at 25 °C. We see that the apparent viscosity of pure 40 mM R_16_HTAB aqueous solution is almost unchanged with the shear rate ($\dot{\gamma }$), suggesting the characteristic of the Newtonian fluid. Furthermore, an extremely low zero-shear viscosity ($\eta_{0}$ = 0.011) indicates that the micellar morphology in the solution is spherical or small ellipsoidal. All mixed solutions exhibit non-Newtonian fluid behavior, steady-state rheology curves of mixed systems reach a Newtonian plateau at low $\dot{\gamma }$, and then shear thinning phenomenon occurs above the critical $\dot{\gamma }$. The shear-thinning is a typical phenomenon of viscoelastic behavior of wormlike micellar solutions, which can be ascribed to the reorientation of the aggregates along the flow direction [29] or the breaking of aggregates into small structures [30] with the shear rate increasing.

All $\eta_{0}$ values of mixed systems are obtained using the Carreau or Cross model by extending the shear rate to zero and are shown in Scheme 2 and Table 1. For the sodium hydroxyl benzoate series, the order of $\eta_{0}$ value is SoHB > SmHB > SpHB. In contrast, the ortho-substituted hydroxyl benzoate (namely salicylate) can vastly induces micellar growth, the $\eta_{0}$ (645.16 Pa·s) of the R_16_HTAB/SoHB solution is five orders of magnitude higher than that of the pure R_16_HTAB solution, indicating the generation of highly viscoelastic wormlike micelles. Whereas the mixed solution with the hydroxyl group in the opposition of benzene ring almost loses its viscoelastic characteristics, and its solution is similar in appearance to the pure 40 mM R_16_HTAB solution; the $\eta_{0}$ is only 0.119 Pa·s, suggesting the micelles are very short. The meta-isomer can induce a small growth in micelles with $\eta_{0}$ of 5.99 Pa·s. The viscosity increasing ability of SB is also much lower than that of SoHB, but is higher than the SmHB and SpHB. The increase in viscosity proves that the aromatic salts have infiltrated into the palisade layer of micelles and interacted with R_16_HTAB, otherwise, the interaction is small. These results are also observed in the mixed systems of CTAB with SoHB, SmHB and SpHB [31,32]. The same results are obtained for the mixed system of three isomers with dodecyltrimethylammonium chloride by microcalorimetry [33]. The mixed solution of three methyl-substituted benzoate derivatives with R_16_HTAB shows the opposite order, with SpMB > SmMB > SoMB. This effect order of the hydrotropes in a cationic surfactant micellar solution agrees with the results reported in the literature for the mixed solutions of the alkyl trimethyl ammonium bromide with ortho-, meta- and para-fluorobenzoate or three isomers of chlorobenzoate [32,34,35]. When both the hydroxyl and methyl groups coexist in the benzene ring of benzoate, the viscosity of the mixed solutions is smaller than when the two groups are alone. However, the result of S4MS is greater than S5MS; similar phenomena have been observed in mixed systems [36].

A large number of studies have confirmed that the high viscosity of mixed solutions is associated with the entanglement of wormlike micelle chains. However, unlike the increase in solution viscoelasticity caused by entanglement of polymer chains, wormlike micelles are in a dynamic equilibrium of continuous breaking and reformation in solution, so they are called living polymers [37,38], and their viscoelastic behaviors show unique characteristics.

### 2.2. Dynamic-State Viscosity of Mixed Solutions

**Figure 3:**
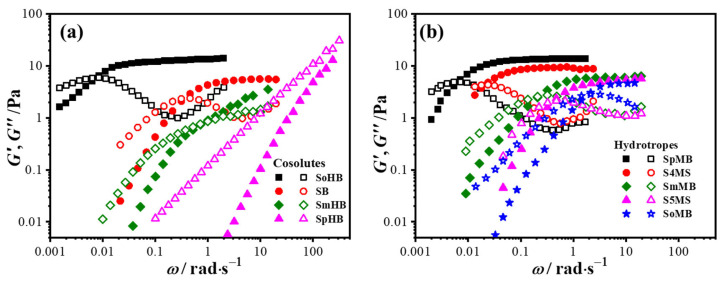
Dynamic moduli as a function of oscillation frequency for the aqueous solutions of R_16_HTAB/ benzoate derivatives (40 mM): (**a**) hydroxybenzoate derivatives, (**b**) methyl benzoate derivative, at 25 °C. The storage shear modulus $G^{\prime }$ (closed circles) and the loss shear modulus G″ (open circles).

The dynamic rheological behaviors of the mixed systems were also investigated by oscillatory shear (frequency sweep). The evolution of the elastic modulus (G″) and viscous modulus (G″) as a function of the frequencies (ω) at a fixed shear stress (σ = 0.1) are shown in Figure 3. The pure R_16_HTAB exhibited no elastic response similar to Newtonian liquids. $G^{\prime }$ and G″ of the R_16_HTAB/SpHB system do not intersect, indicating that the micelles are relatively short and are not entangled. Other mixed solutions displayed a typical viscoelastic response, which is probably due to entanglement of wormlike micelles in varying degrees to form a transient network. The $G^{\prime }$ is lower than the G″ in the low shear frequency region or long time scales; the systems showed a viscous behavior (G″ > $G^{\prime }$), but then it crosses each other at a critical frequency $\omega_{c}$ (where $\omega_{c}=1/\tau_{R}$). At higher *ω*, the system showed more elastic behavior ($G^{\prime }$ > G″). These changes in $G^{\prime }$ and G″  with $\omega_{c}$ reflecting the viscoelastic behavior of wormlike micelles can be described by a Maxwell fluid model, as follows: [39,40]:

(1) $$G^{\prime }=\frac{{\left(\omega \tau_{R}\right)}^{2}}{1+{\left(\omega \tau_{R}\right)}^{2}}G_{0}$$

(2) $$G^{\prime \prime }=\frac{\omega \tau_{R}}{1+{\left(\omega \tau_{R}\right)}^{2}}G_{0}$$

where *ω* and *τ*_R_ are the angular frequency and relaxation time, respectively, and the value of *τ*_R_ can be obtained by the reciprocal of the intersection ($\omega_{c}$) of the angular frequency, where the moduli G″ and $G^{\prime }$ overlap. $G_{0}$ is the platform value of $G^{\prime }$ at high frequency. The Cole–Cole plot (plot of G″ as a function of $G^{\prime }$) can be used to estimate whether the data fit the Maxwell model, which reveals a semicircular shape as follows:

(3) $$G^{\prime \prime }+{\left(G^{\prime }-\frac{G_{0}}{2}\right)}^{2}={\left(\frac{G_{0}}{2}\right)}^{2}$$

Kern et al. [41,42] recommend that $G_{0}$ can also be obtained by extrapolation from the Cole-Cole graph, that is, the data points deviating from the semi-circle can be extrapolated to the horizontal axis, and the corresponding value is $G_{0}$. When $G_{0}$ of the system cannot reach a constant limit value, Acharya et al. [43,44] suggested that $G_{0}$ could be estimated from the viscous modulus (expressed as $G_{min}^{\prime \prime }$) at shear frequency $\omega_{c}$, namely $G_{0}=2G_{min}^{\prime \prime }$.

In Figure 3, the values of corresponding parameters of wormlike micelles calculated from fitting the rheological curves are given in Table 1. The value of $\tau_{R}$ for R_16_HTAB /SpHB solution is too short to be detected, so $G_{0}$ is not going to be available. It can be observed that the law of evolution of both the $\tau_{R}$ and $G_{0}$ is consistent with the $\eta_{0}$ for different mixed solutions. This is because $\tau_{R}$ is associated with the contour length of wormlike micelles (*L*_c_) [44]. An increase in the *τ_R_* can be related to the growing micelles leading to longer ones, followed by the occurrence of entanglement phenomenon, which makes the solution stickier. Namely, the larger the *τ_R_* value is, the slower the relaxation and diffusion of wormlike micelles and also the longer the micellar size, and vice versa. The $G_{0}$ usually depends on the number density of the aggregates and therefore reflects the mesh size of the network. The increase in the plateau modulus $G_{0}$ corresponds to the increase in the degree of entanglements, which can be regarded as evidence to prove the micelles grow linearly, leading to the increase of the contour length of the micelles.

In Table 1, the $G_{min}^{\prime \prime }$ the minimum value of G″ at the high-frequency region to $G_{0}$, and the $L_{c}$ is the average contour length of the micelles. For wormlike micelle solutions with Maxwellian behavior, the relationship between them follows the relation [45]

(4) $$\frac{G_{0}}{G_{\min}^{\prime \prime }}\approx \frac{L_{c}}{l_{e}}$$

where $l_{e}$ is the average length between two entanglement points and a value of 80–150 nm for wormlike micelles was adopted [44]. The results of the calculation show that the $L_{c}$ corresponds to roughly 0.4–0.8 μm for R_16_HTAB/SB mixed system, 0.2–1.9 μm for R_16_HTAB/sodium benzoate systems with different hydroxyl substituent positions, 0.2–3.5 μm for R_16_HTAB/sodium benzoate systems with different methyl substituent positions and 0.4–1.8 μm for both the R_16_HTAB/S5MS and R_16_HTAB/S4MS systems. From the variation tendency of rheological parameters, it can be judged that SoHB has the strongest ability to promote the growth of micelles, followed by SmHB and SpHB for sodium hydroxyl benzoate series. While for the sodium methyl benzoate series SpMB showed the greatest ability, which are consistent with the variation tendency in the $\eta_{0}$. In a nutshell, the $\eta_{0}$, $\tau_{R}$, $G_{0}$, and $L_{c}$ decrease in the following order: SpMB > SoHB > S4MS > SmMB > S5MS > SB > SmHB > SoMB > SpHB, which is in correspondence with the macroscopic observation.

**Figure 4:**
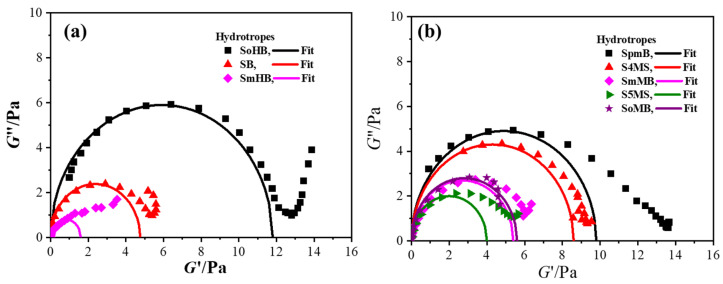
Cole–Cole plot of R_16_HTAB/ benzoate derivatives (40.0 mM): (**a**) hydroxybenzoate derivatives, (**b**) methyl benzoate derivative at 25 °C; solid lines indicate the best fitting of the Maxwell model.

Figure 4 shows a Cole–Cole plot with the $G^{\prime }$ and G″ data extracted from Figure 3, where the solid lines correspond to the Maxwell evolution. At low and medium frequency range, the trend of the curves accords with the Maxwell fluid behavior, whereas at high frequency, the results diverge from the semicircular plots. This is a characteristic of wormlike micelles [46] because the micelles are in a state of instantaneous equilibrium where they are constantly being reorganized and destroyed and the high shear rate can speed up the breaking and recombination of the wormlike micelles. Hence, *t*_break_ (breaking time of micelles) increases, leading to the deviation from the Maxwell phenomenon.

### 2.3. Microscopic Morphology of Mixed Micelles and Mechanism Discussion

**Figure 5:**
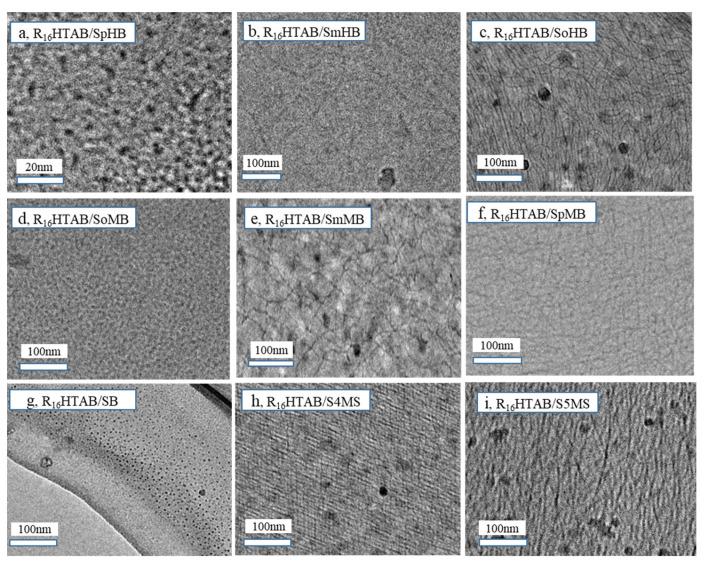
The representative Cryo-TEM images of 40 mM R_16_HTAB/40 mM benzoate derivatives mixed solutions.

Macroscopic appearance of R_16_HTAB/hydrotrope solution with viscosity variations in Figure 1 can be explained by wormlike micelles variations induced by different hydrotropes. Cryo-TEM images in Figure 5 show varied micellar microstructures in different mixtures. Similar with R_16_HTAB/SB mixture, spherical and ellipsoidal micelles are observed in the solution of R_16_HTAB/SpHB (Figure 5a,g). Figure 4b reveals the presence of longer wormlike micelles in the R_16_HTAB/SmHB solution. Extremely long wormlike micelles are clearly observed in the R_16_HTAB/SoHB system. The majority of them is overlapped and entangled to form a 3D network (Figure 5c), resulting in a significant increase in viscosity of the solution. Interestingly, compared with the above results (Figure 5a–c), we found that the density of wormlike micelles is decreased when the corresponding hydroxyl group was replaced by methyl group (Figure 5d,f). Moreover, the number of wormlike micelles in R_16_HTAB/methylbenzoate anions is strongly dependent on the relative position of the methyl and carboxyl groups and is conversely ranked in the order of p-, m-, and o-methylbenzoate anions compared with R_16_HTAB/hydroxybenzoate anions (Figure 5d,f). Additionally, the co-existence of methyl and hydroxy groups apparently accelerating the growth of spherical micelles into wormlike ones. Only a small amount of the wormlike micelles were tangled together in the R_16_HTAB/S5MS system (Figure 5h). However, the majority of long wormlike micelles are arranged densely and orderly, which are overlapped and entangled with each other in the R_16_HTAB/S5MS system (Figure 5i). These threadlike micelles are spontaneously formed in the above mixtures with average diameters of 3 nm and 40 μm in length.

To the best of our knowledge, the effect of organic salts on micellar transition is much more complex than inorganic ones. They are influenced by the following characteristics of the hydrotropes:

1. The presence of a hydroxyl group or methyl group.
2. The hydrophilic and hydrophobic properties of substituent groups, and the position and orientation of the substituent groups on the benzene ring.
3. The size, polarization and hydration of the substituent groups
4. The ability of substituent groups to form hydrogen bonds with the R16HTAB and their electrical properties.
5. The molecular structure characteristics of R16HTAB.

Based on the concept of molecular packing parameter proposed by Israelachvili et al. [1], spherical micelles can form spontaneously from amphiphilic molecules whose packing parameter (*P*) is less than 1/3, wormlike micelles can form when P is between 1/3 and 1/2, where *P* = *v*_0_/*al*_0_, *v*_0_ is the surfactant tail volume, *l*_0_ is the tail length, and *a* is the occupied area per amphiphile at the aggregate surface.

After adding the cosolvents to the surfactant solution, the anion of additives binds strongly to the surfactant cation. The electrostatic effect reduces the repulsion between cationic head groups of R_16_HTAB, thus reducing the *a*. With the increase in the *P*, the micelle morphology follows the change rule from spheroidal micelles to wormlike (rod-like) micelles.

**Figure 6:**
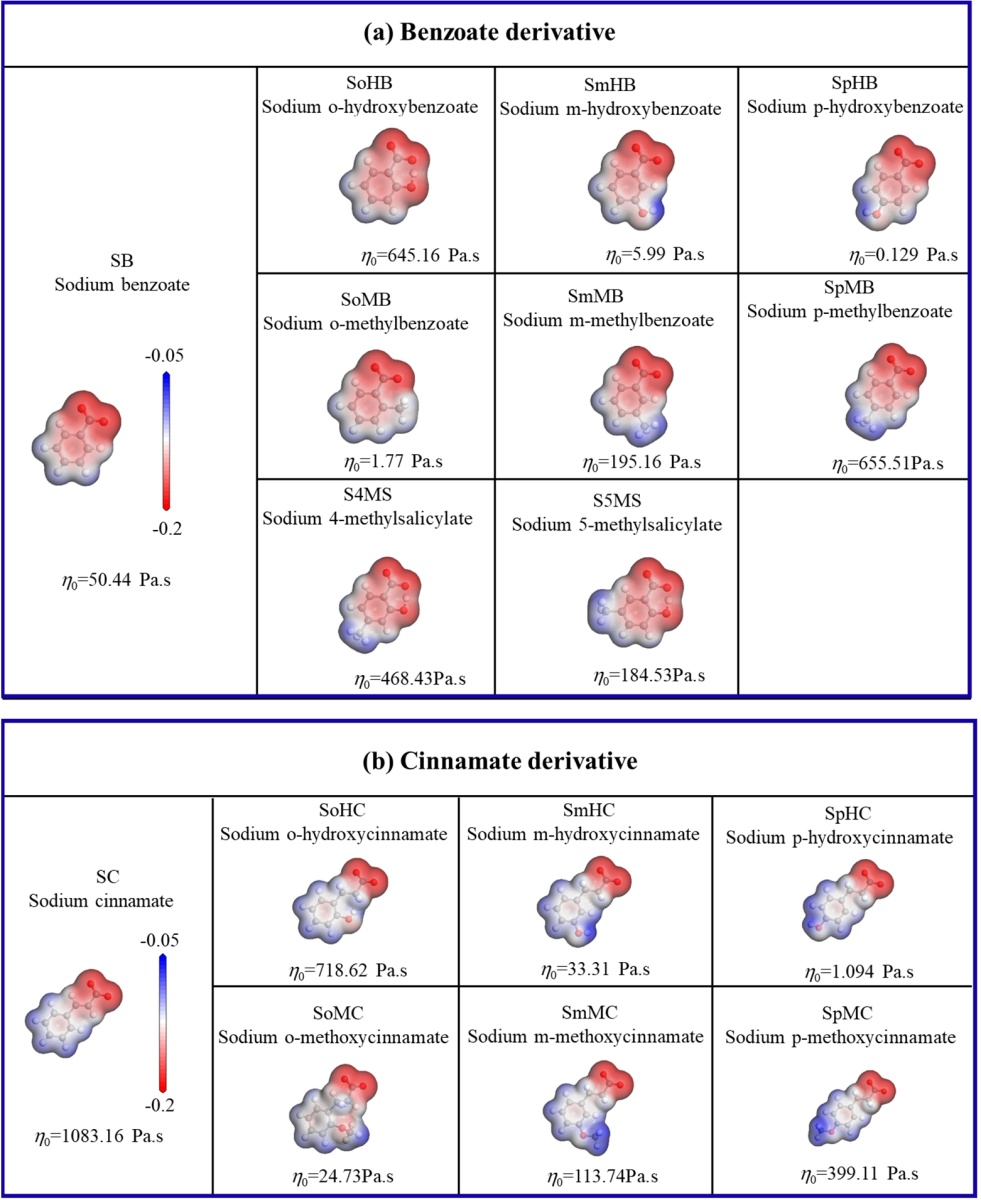
The molecular electrostatic potential of (**a**) benzoate derivatives and (**b**) cinnamate derivatives.

To better understand the effect of hydrotropes with various structures on the electrostatic interactions with R16HTAB, the density functional theory (DFT theory) was used to calculate the electrostatic potential of hydrotropes shown in Scheme 2 (Figure 6). The red region corresponds to electronegativity where the color saturation equals to the intensity of electrostatic potential. The order of molecular electrostatic potential is SoHB, SpHB and SpHB in Figure 6a. Therefore, SoHB has the strongest ability to reduce the effective headgroup area of cationic surfactant, thereby greatly promoting the growth of wormlike micelles.

**Figure 7:**
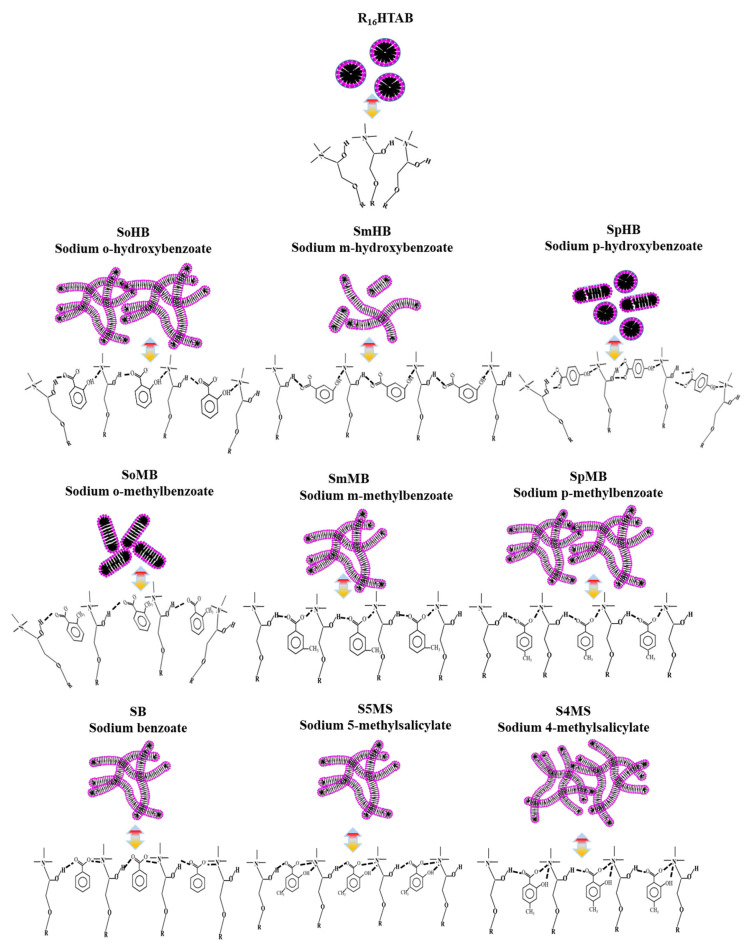
The schematic representation of microstructure transformation and proposed mechanisms for the mixed micellar solutions with different rheological properties induced by different substituents. The arrows correspond to the different packing state of wormlike micelles.

From the geometric point of view (Figure 7), the SoHB molecule stands upright on the interface of the micelle, which induces viscoelasticity in R_16_HTAB; the SmHB molecule tilts toward the surface of the micelle and it is not conducive for micellar growth, but forms short micelles. However, the molecule SpHB, which does not induce viscoelasticity, orients with its carboxylic group tilts horizontally on the surface of the micelle, which increases the spatial volume of the micelle surface, and hence micellar formation is difficult, viscoelasticity is not observed. The order of the spacer distance between the carboxyl group and the methyl group for the methyl -substituted benzoate derivatives is SpMB > SmMB > SoMB. Therefore, we speculate that the order of the effective headgroup area of surfactant in three systems is SpMB < SmMB < SoMB, since the steric hindrance of methyl group in SoMB is much larger than SpMB, hindering the close packing of the surfactant micelles, resulting in a rapid decrease in solution viscosity. There is no significant difference between the molecular electrostatic potential of S4MS and S5MS from Figure 6. However, the interaction between S4MS and R_16_HTAB is apparently stronger because the intermolecular hydrogen bonds and the electrostatic interaction forces between the molecules work synergistically, as depicted in Figure 7.

The possible mechanism we obtained could also be used to explain the interaction between R_16_HTAB and hydrotropes displayed in Scheme 2b. The hydrogen bonding possibly could be formed between hydroxy group in SoHC but hardly with SpHC since the hydroxy group in SoHC is too difficult to interact with carboxyl group due to the long distance between them. The order of steric hindrance of methoxyl-substituted cinnamate derivatives is SoMC > SmMC > SpMC, hindering the electrostatic interaction with R16HTAB. Therefore, the intensity of viscosity shows the following trends: SoMC < SmMC < SpMC.

## 3. Materials and Methods

### 3.1. Materials

3-hexadecyloxy-2-hydroxypropyltrimethylammonium bromide (R_16_HTAB, Scheme 1) was synthesized by the same method of our earlier work [26], and aromatic hydrotropes (Scheme 2) were obtained from Shanghai Chemical Co., Ltd., Shanghai, China and Sigma-Aldrich Reagent Co., Shanghai, China. The additives purchased in acid form were obtained by neutralizing the respective pH with sodium hydroxide (NaOH, ≥99%) solutions, followed by the lyophilization of the solvent. Sodium benzoate (SB, 99.5%), sodium o-hydroxybenzoate (SoHB) and sodium m-hydroxybenzoate (SmHB) and sodium p-hydroxybenzoate (SpHB) (99%); o-methylbenzoate (99%); m-methylbenzoate and p-methylbenzoate (98%), 4-methylsalicylate and sodium 5-methylsalicylate with purity 99%. Sodium cinnamate (SC) and 2-hydroxycinnamic acid (98%), 3-hydroxycinnamate and 4-hydroxycinnamate (99%), o-methoxycinnamate and m-methoxycinnamate (99%), p-methoxycinnamate (>98%). Ultrapure deionized water for all experiments was prepared by re-distilling deionized water upon dealing with potassium permanganate.

### 3.2. Sample Preparation

Mixtures of solid cationic surfactants R_16_HTAB (80 mM) and organic anions (80 mM) were prepared by dissolving certain amounts of each component separately and adding progressively one solution (1.5 mL) to the other one (1.5 mL) in a water bath and swirled magnetically. In this case, an equimolar system of R_16_HTAB/hydrotrope was prepared. The prepared solutions were maintained at 25 °C for at least 5 days to achieve equilibrium.

### 3.3. Rheological Measurements

The rheological measurements of solutions were performed using an MCR 302 rheometer with a cylindrical pp 50 or 25 mm rotor (Anton Paar, Graz, Austria). A centrifuge (3000× *g*, 5 min) was used to expel air bubbles in the samples before the measurements. The gap value between rotor and Peltier was 0.5 mm. The rheometer was installed with a Peltier plate, which can insure the precise control of the temperature (uncertainty: 25 ± 0.05 °C). The dynamic sweeps were achieved at a fixed stress (selected in the linear range). All operations were repeated twice for pledge reproducibility.

### 3.4. Cryogenic Transmission Electron Microscopy

The specimens for the Cryo-TEM measurement were produced in a controlled environment vitrification system. A 5 μL solution was dripped onto a copper grid. Then, the mesh pores were smeared using two pieces of filter paper to generate a thin film. The specimen was fleetly dropped into a liquid ethane tank at −165 °C after a few seconds. The vitrified copper grid was carefully placed into a cryogenic sample rod and observed at 120 kV with a JEM-1400 TEM (JEOL, Tokyo, Japan) and a Gatan US1000 894CCD monitor (JEOL, Tokyo, Japan).

## 4. Conclusions

In summary, the interaction between R_16_HTAB and 16 types of hydrotropes with various structures was comprehensively studied. The spacer distance between the carboxyl group and the hydroxyl, methyl or methoxyl group greatly affect the electrostatic interaction and the accessibility of hydrogen bonding formation. Ortho-hydroxybenzoate or cinnamate derivative has the strongest interactions with R_16_HTAB, whereas para-methyl (methoxyl) benzoate or cinnamate derivative show contrary trends. A possible mechanism was proposed by analyzing the zero-shearing viscosity (η_0_), the apparent viscosity and the possible interactions between molecules including electrostatic interaction, hydrophobic and hydrogen bonding. The strong electrostatic interaction and the formed intermolecular hydrogen bonding probably reduce the interface curvature of spherical micelles and leading to one-dimensional growth of the micelle, thereby facilitating the transformation of the spherical micelle into a rod or wormlike shape. The micellar entanglement occurs and the viscosity of the solution increases. We hope these results can be promising to directionally enrich the designing and preparation of smart wormlike micelles and thus extending their application in different fields.

## Figures and Tables

**Table 1:** Rheological parameters of wormlike micelles with different benzoate derivatives, R_16_HTAB/ benzoate derivatives = 40/40 mM.

Hydrotropes	η_0_ (Pa·s)	ω_c_ (red/s)	τ_R_ (s)	G_0_ (Pa)	G”_min_ (Pa)	L_c_ (nm)
SB	50.44	0.388	2.58	5.42	0.97	437–821.2
SoHB	645.16	0.008	125.0	12.6	0.92	1018.2–1919.1
SmHB	5.99	0.869	1.15	1.69	1.09	113.6–213.0
SpHB	0.129	-	-	-		
SoMB	1.77	2.551	0.392	4.63	1.65	224.5–420.9
SmMB	195.16	0.251	3.98	6.43	1.08	476.3–893.1
SpMB	655.51	0.006	161.29	14.52	0.63	1843.8–3457.1
S4MS	468.43	0.021	47.62	9.66	0.79	978.2–1834.2
S5MS	184.53	0.286	3.49	5.54	1.08	410.4–769.4

## Data Availability

The datasets used and/or analyzed during the current study are available from the corresponding authors on reasonable request.
